# Selected Articles

**Published:** 1855-10

**Authors:** 


### Selected Articles.

### TREATMENT OF ALVEOLAR ABSCESS.

BY PROF. WHITE.

From my Note Book.—Master G——, 14 years of age, good constitution, applied to us in the summer of 1846, suffering from severe abscess of both the superior front incisor teeth, in which the nerves had been destroyed for some time by a blow, and which had also broken off the median angles sufficiently to almost explore the nerve cavities. The teeth were extremely loose and discharging pus at several points around their necks, between the gums and the teeth; so much were the teeth loosened by the suppuration that it was feared they would drop from their sockets; we were desired to extract them as it was not believed that they could possibly be saved by any treatment. We lanced the gum at two points, about half an inch above the margin, opposite the ends of the roots and deep enough to meet the pus sacks, into which we inserted tents of cotton in order to effect a drainage of the pus in a new direction, so that the gums might contract and heal, or grow to the necks of the teeth again, if they had not lost their vitality entirely; our expectations were fully realized; in a few days the pus ceased discharging around the necks of the teeth, and escaped entirely through the openings which we had made in the gum. The gums grew firmly to the teeth, and the swelling subsided rapidly, and in a few weeks the tents were left out. These tents of cotton were renewed every other day. They were made by rolling a small portion of loose cotton between the fingers, making one end larger than the other, a kind of bullet-headed roll; the larger end was introduced into the opening a considerable distance, and the smaller or tail end remaining out about a quarter of an inch, so as to afford a hold by which to withdraw them when desired, and to prevent the lips of the wound from closing. In this way a complete drainage of the parts is constantly kept up upon the principle of the *seton* until all irritation is reduced, and the *piogenic* membrane over the apexes of the roots is entirely absorbed, when upon the withdrawal of the tents the sinuosities *may*, and they generally *do* heal entirely. At this time, when the gums had healed, the pulp cavities were both opened from the fractured surfaces, which showed some signs of decay; the internal walls of the cavities were also considerably decayed; they were thoroughly cleansed, and plugged to the apexes of the fangs with gold. They have not given the slightest pain or sign of abscess up to this time. The gums present a slightly blue appearance, but the teeth are as firm in their sockets as any teeth in the mouth. The teeth seem to have *less* motion than is usual in health, from the fact that there seems to be less vascularity about the parts than is usual in more healthy teeth. There is no thickening of the gums, but rather an impoverished look. This method of treatment presented itself to our mind in the spring of 1839, when we first resorted to it in treating two front teeth roots for a surgeon in the United States Navy, and which was entirely successful. The abscesses never returned, but the roots were removed within a year past, from having become too much decayed to hold pivot teeth. These cases furnish several important and exceedingly interesting considerations in the treatment of alveolar abscess; and first, that of diverting the discharge of the pus by a new channel to save the life of the tooth. We are treating two cases of the kind at present, and have treated a very great number from time to time. Secondly, maintaining the mouth of the wound or orifice open, so as to favor the constant escape of the pus as rapidly as it is secreted in the sack, and concentrating it to one point, and preventing burrowing through surrounding parts, and of obtaining the advantage of capillary attraction through and around the tent of cotton, in the same way that the drainage of abscesses is accomplished by the surgeon it other parts of the body; such as in the treatment of scrofulous abscesses of the scalp. Again, how will the re-union of the gums to the necks of the teeth be explained by those who contend that the teeth become foreign bodies when their pulps are destroyed? In these cases the pulps were entirely dead and the external membranes of the roots for a time nearly dissected away by the escape of pus. Some of our eminent surgeons are in the habit of lancing abscesses of the gums every day for a long time, from the fact that the wound heals as soon as the tension of the parts is relieved by the first discharge of pus; of course a tent of cotton should always be introduced into the wound in lancing any abscess.

### HIGHLY INTERESTING TO DENTISTS.

Discovery of a new mode of producing anæsthesia, by ALEXANDER FLEMING, M. D., Professor of Materia Medica, Queens College, Cork, by compression of the carotids.

The mode of operating is, "to place the thumb of each hand under the angle of the lower jaw and feeling the artery, to press backward and obstruct the circulation through it. The recumbent position is the best, and the head of the patient should lie a little forward, to relax the skin. There should be no pressure on the windpipe." * * * * "There is felt a soft humming in the ears, and a sense of tingling steals over the body, and in a few seconds complete unconsciousness and insensibility supervene, and continue as long as the pressure is maintained. On its removal there is confusion of thought, with a return of the tingling sensation, and in a few seconds consciousness is restored. The operation pales the face slightly, but the pulse is little if any at all affected. In profound sleep the breathing is stertorous, but otherwise free.

The experiments have never caused nausea, sickness, or any other unpleasant symptom, except in a few instances slight languor. The period of profound sleep in my experiments have seldom exceeded fifteen seconds, never half a minute."

We have tried this as directed above, and have succeeded in producing entire somnolence, at least, and have good reason to suppose, anæsthesia, for we have performed several small but painful operations under its influence with the same indications of its being painless, as is shown under the inhalation of ether or chloroform. The Profession will doubtless soon test its value and give us results, and we refrain from further remarks, hoping in the next No. of the Obturator to have a collection of interesting facts to record.—*Dental Obturator.*

---

### MANAGEMENT OF LIGHT IN THE PERFORMANCE OF DENTAL OPERATIONS.

BY. J. M'QUILLEN, D. D. S.

In the course of a recent conversation with a fellow practitioner, he remarked: "Ten years from now you will find it necessary to operate by a stronger light than the northern."

With the best reasons in the world for doubting the correctness of this assertion, I am willing, for the sake of argument, to suppose his position to be a tenable one, and then, in reply, would ask whether it is any more advisable to at once adopt the intense and variable southern light, than it would be for those persons who are compelled to use spectacles, to have lenses of the highest power introduced in the beginning; because, convinced that, at some period in life's journey, it will be necessary to employ them, to secure perfect vision.

The premature adoption of either would, under such circumstances, but become an additional cause in impairing the usefulness of the organ.

Dr. Kitchner, in his "*Economy of the Eyes*," says, that he was about fifteen years old when he first discovered that he could not discern distant objects so distinctly as people who have common eyes usually do. "Seeing," he remarks, "that I could not see what persons with common eyes frequently pointed out to me as well deserving my attention, I paid a visit to an optician, and purchased a concave eye-glass, No. 2. After using this some little time, I accidentally looked through a concave No. 3, and finding my sight much sharper with this than with No. 2, had my spectacles glassed with No. 3, which appeared to afford my eyes as much assistance as they could receive. After using No. 3 for a few months, I chanced to look through No. 4, and again found the same increase of sharpness, &c., which I perceived before, when I had been using No. 2 and first saw through No. 3, therefore concluded that I had not yet got glasses sufficiently concave, and accordingly procured No. 4; however, this soon became no more stimulus to the optic nerve than its predecessors, Nos. 2 and 3, had been. I then began to think that the sight was subject to the same laws which govern the other parts of our system, *i. e. an increased stimulus, by repetition, soon loses its power to produce an increased effect.* Therefore, I refused my eye any further assistance than it received from spectacles glassed with No. 2, which I have worn for upwards of thirty-one years, and it is very nearly, if not quite, as sufficient help to me now, as when I first employed it."

In large cities where the owners of property care more about taking advantage of every foot of ground for building purposes, than they do about the proper ventilation of, and introduction of light to the tenements they erect; the dentist, though possessing an excellent northern light, frequently, if not invariably, finds the limited ground back of his office, hemmed in on two, and sometimes three sides, by back buildings two stories high. The red light reflected from the surrounding brick walls into the room, is a source of annoyance to the operator on account of its being an unnatural and cross light, to that which is transmitted to the sky, and thus impairing its purity and usefulness.

To overcome this difficulty, an intimate friend had a portion of the wall of his back building, and the side of a house, in the rear of the lot, painted green. Though this is preferable to the red brick, a less decided color would be more attractive to the eye, at the same time that it is better adapted to the purpose.

Those quiet shades, called by artists neutral tints, such as drab, grey, fawn, or brown, seem to me to be the most appropriate colors that can be selected. The window shutters, and fence separating the yard from the next doors neighbor's, should be painted of the same shade as the walls. White, the usual color, is, without exception, the most objectionable one that can be used.

This plan is the best one to pursue, where it is impossible to have the walls covered by an ivy. Even where the latter is admissible, these shades could be applied so as to obviate the reflection from the red bricks, until such time as it shall have grown sufficient to cover the entire wall.

The property possessed by the vegetable kingdom, of *absorbing* a large portion of light and *reflecting* very little, makes the ivy the most desirable covering that can be obtained for the walls. At the same time that it is much more pleasant to look at than bare bricks, the eye of the patient can rest upon it during the entire sitting, without a sense of fatigue; the associations connected with it, likely to spring up in the mind, carrying the thoughts far from the unpleasant operations submitted to, it is not necessary to dwell upon.

Three different kinds of ivy have been shown to me by gardners with whom I have conversed on this subject. They are the American, European and Giant. Of these the former is the most luxuriant and rapid in its growth, but owing to the falling of its leaves in the autumn, it is not adapted to our purposes. The European or evergreen will grow in shady localities, and retains its dark green foliage throughout the entire year; its growth, however, is quite slow. The Giant ivy, on the contrary, grows rapidly, is much more vigorous, and possesses a larger and richer leaf, which is also retained at all seasons of the year. These advantages make it the most desirable one that can be selected to overcome the difficulty in question.

The objection that is urged against the ivy, of ruining the wall to which it is attached, is without foundation; it is, on the contrary, a preservative. And as an evidence, I would cite the time-honored ruins of England, that are overrun by it.

In conclusion, I offer the following suggestions, as being worthy of some attention.

When operating upon cavities situated in the palatine surface of superior incisors and canines, and the posterior approximal surfaces of molars or bicuspids, either in the upper or lower jaw, every operator has no doubt frequently found some difficulty in getting sufficient light on the surface demanding attention, to obtain a perfect view of the cavity, owing to the shadow cast by the tooth operated on. The *stronger* the light, the more decided the *shadow*. To obviate this, when operating on the upper front teeth, I have found a small piece of white muslin, folded so as to cover the tongue, but not protrude from the mouth, of decided service. The light striking upon the muslin, is reflected to the palatine surface of the teeth, and defines the margins of the cavity perfectly. If the cavity is situated in the posterior approximal surface of a molar or bicuspid, a still smaller piece of muslin, held in place by the forefinger back of the tooth operated on, will reflect sufficient light to give a perfect view of the cavity.

To prevent abrasion of the cuticle at the corners of the mouth, it was my habit for years, as it has been that of many operators, to protect the skin, by napkins, from coming in contact with the shaft of the instrument. For the last three years, however, I have discontinued the practice, from a conviction that the maintenance of such a course would be subjecting my eyes to a most injurious influence.

Owing to the napkin being white, none of the rays of light that fall on it are *absorbed*, but all are *reflected* directly into the eye of the operator.

It is quite common for seamstresses to complain of a sense of weariness and pain in their eyes, after they have been engaged continuously for two or three weeks on white work; and it is not an unfrequent occurrence, that they are compelled to seek the advice of the occulist, to obtain relief.

Though the surface of white offered to the eye of the dentist, is quite limited, yet, like water falling drop by drop on the stone, it will eventually make a perceptible impression.

I will acknowledge candidly, that I am not sufficiently of a self-sacrificing nature, to be willing to spare my patients a slight and *temporary* excoriation of the cuticle, at the risk of inflicting a *permanent* injury on my eyes.

These objections will not bear against the use of the muslin for throwing light into cavities, described above. That is only required occasionally, whereas the napkin is used continually.—*Dental News Letter.*

